# Mechanism of mitotic recombination: insights from *C. elegans*

**DOI:** 10.1016/j.gde.2021.06.005

**Published:** 2021-12

**Authors:** Ondrej Belan, Roopesh Anand, Simon J Boulton

**Affiliations:** DSB Repair Metabolism Laboratory, The Francis Crick Institute, London NW1 1AT, UK

## Abstract

Homologous recombination (HR) plays a critical role in largely error-free repair of mitotic and meiotic DNA double-strand breaks (DSBs). DSBs are one of the most deleterious DNA lesions, which are repaired by non-homologous end joining (NHEJ), homologous recombination (HR) or, if compromised, micro-homology mediated end joining (MMEJ). If left unrepaired, DSBs can lead to cell death or if repaired incorrectly can result in chromosome rearrangements that drive cancer development. Here, we describe recent advances in the field of mitotic HR made using *Caenorhabditis elegans* roundworm, as a model system.

**Current Opinion in Genetics & Development** 2021, **71**:10–18

This review comes from a themed issue on **Mechanisms of Homologous Recombination**

Edited by **Eric C Greene** and **Rodney Rothstein**

For a complete overview see the Issue and the Editorial

Available online 26th June 2021

**https://doi.org/10.1016/j.gde.2021.06.005**

0959-437X/© 2021 The Authors. Published by Elsevier Ltd. This is an open access article under the CC BY license (http://creativecommons.org/licenses/by/4.0/).

## *Caenorhabditis elegans* roundworm: an unconventional, but uniquely useful model to study mitotic recombination

The nematode is a particularly attractive model to study HR due to several key aspects of this organism. Firstly, the small size and short life cycle of nematodes permits the study of HR genes during development and aging. Secondly, the spatial organisation of the germline, with mitotic cells located at the distal tip followed by progressive stages of meiosis I, facilitates the simultaneous evaluation of HR factors in different cell types and meiotic stages. Thirdly, reverse genetic manipulation by RNAi or CRIPSR-Cas9 are particularly robust [1,2] and can be readily delivered by feeding or injecting into the germline allowing detailed phenotypic screening and analysis. Furthermore, the deletion of many HR genes that are lethal in mammalian cells are tolerated in the nematode system [3, 4, 5], which provides a means to study HR deficiency at a whole-organism level. Finally, compared to mammalian systems, many nematode HR proteins are readily amenable to biochemical characterization due to their smaller size and optimal activity at room temperature [6].

Protein–protein interaction mapping and genetic approaches were instrumental in drafting a basic map of the *C. elegans* DNA damage response (DDR) [7]. Following the identification of candidate DDR genes, diverse methodical approaches to study many unique aspects of HR in *C. elegans* have been developed over the past 15 years. The monitoring of larval growth after exposure to DNA damage can be used to assess the DNA damage sensitivity in different genetic backgrounds. Similarly, the accumulation of DDR factors at sites of DNA damage by immunofluorescence is routinely employed to study the response in different cell types and developmental stages. Importantly, live-worm imaging can provide detailed kinetics of recruitment of critical DDR factors such as RAD-51 and RPA [8] in perturbed conditions. More recently, the development of super resolution microscopy techniques such as structural illumination microscopy (SIM) has allowed the visualization of recombination intermediates in the nematode germline despite its thickness along the z-axis [9^••^]. In terms of monitoring recombination upon genetic or pharmacological intervention, well-established fluorescent HR reporters such as DR-GFP in mammalian cells, have been adapted to monitor HR efficiency in *C. elegans* [10]. Recently, the application of whole-genome sequencing of worms has been used to understand the nature and origin of genomic scars in a panel of HR-deficient *C. elegans* mutants [11^••^,12^••^]. Finally, given the relative feasibility of obtaining soluble recombinant nematode HR factors, the key steps of HR pathway such as strand invasion [13] and single-strand annealing [6] have been successfully reconstituted *in vitro*. Collectively, the development of these assays has paved the way for advanced structural and biophysical investigation of HR [14^••^,15^•^], which has been particularly challenging with the mammalian proteins.

## Nematode mitotic HR: canonical DSB repair step by step

### DNA end resection and DNA damage signalling

DSBs can either occur directly following exposure to certain DNA damaging agents or may arise as intermediates of various DNA repair processes such as DNA inter-strand crosslink (ICL) repair. Depending on the nature of the damaging agent, DSBs can be either single-(replication block) or double-ended DSBs (i.e. ionizing radiation).

**Figure 1:**
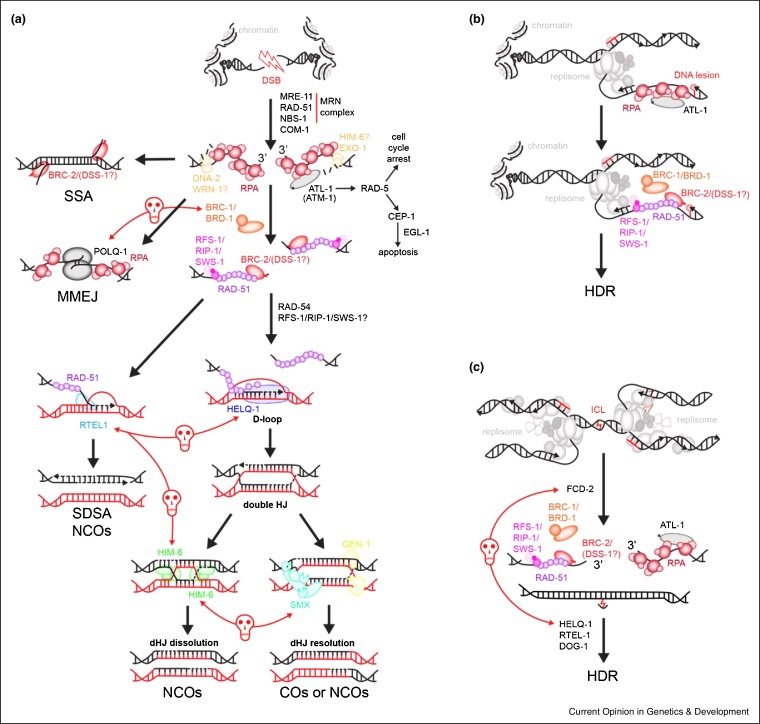
Overview of mitotic homology-directed repair pathways in *C. elegans*. **(a)** Canonical DSB repair HR pathway in *C. elegans*. DSB — DNA double-strand break. SSA — single-strand annealing. Key proteins described in the text are shown with correspondingly coloured text. Key HR subpathways are marked: SSA — single-strand annealing, MMEJ — microhomology-mediated end-joining, SDSA — synthesis-dependent strand annealing. Key HR intermediates: D-loop — displacement loop and dHJ — double Holliday junction are marked. Recombination products, NCOs — non-crossovers and COs — crossovers, are formed either through SDSA, dHJ dissolution by HIM-6, or dHJ resolution by HJ resolvases. SMX stands for SLX-4/SLX-1/MUS-81/XPF-1 resolvase complex. Red lines with red skulls represent synthetic lethal interaction/parallel pathways as referred to in the main text. **(b)** Early steps of recombination at damaged replication forks. HDR — homology-directed repair. **(c)** Homologous recombination acting downstream of FCD-2 in repair of DNA inter-strand crosslinks (ICLs). HDR — homology-directed repair. Red lines with red skulls represent synthetic lethal interaction.

During classical double-ended DSB repair (Figure 1a), DNA breaks are sensed and processed by the MRN complex, consisting of MRE-11, RAD-50 and the recently identified NBS-1 [16^•^]. This complex is critical for DNA end resection, which entails the excision of single strand in 5′ to 3′ direction. COM-1, a nematode homolog of human CtIP [17], also participates in DNA resection, but unlike in other systems, COM-1, MRE-11 and NBS-1 also suppress CKU-70 mediated end-joining at DNA breaks [16^•^,^18^,^19^]. The 3′ ssDNA overhang generated by DSB resection is further resected by DNA-2 [20] and EXO-1 [16^•^]. Whether HIM-6 (homolog of mammalian BLM helicase) or WRN-1 play a role in end-resection in *C. elegans* remains unknown. RPA bound ssDNA also recruits and activates ATL-1 (ATR homolog). ATL-1, aided by ATM-1 (homolog of human ATM kinase), triggers downstream signalling, with RAD-5 (homolog of human TEL2) promoting cell cycle arrest. In cases of persistent DNA damage, the p53 homolog CEP-1 can also trigger apoptosis through induction of EGL-1 [21].

## BRCA–RAD51 axis of presynaptic filament assembly

**Figure 2:**
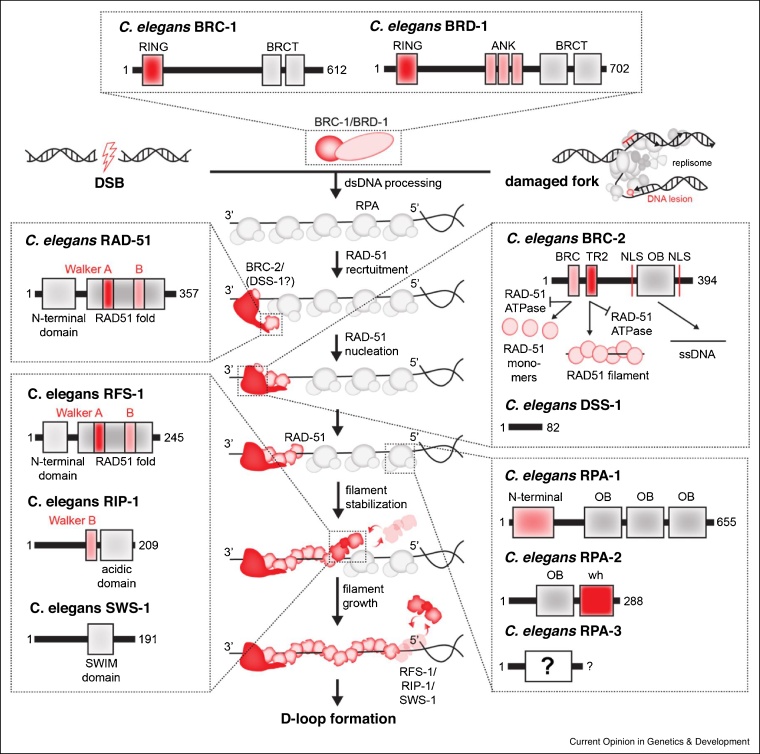
Factors involved in formation of RAD-51 presynaptic filament. BRC-1 — BRCA1 homolog. BRD-1 — BARD1 homolog. Both proteins act in upstream signalling pathway and/or possibly downstream to promote RAD-51 loading/stabilization. BRC-2 — BRCA2 homolog acts possibly in conjunction with DSS-1 to recruit and nucleate RAD-51 clusters to ssDNA and promote replication protein A (RPA) displacement. These clusters are stabilized via TR2 domain of BRC-2. The growth of RAD-51 filament from initial nucleating events helps to form nascent filaments. These are bound and stabilized by RFS-1/RIP-1/SWS-1 (RAD-51 paralog-Shu) complex. ATPase activity of RFS-1 allows the complex to dissociate to allow iterative cycles of stabilization and growth. This ‘proof-reads’ RAD-51 into long filaments capable of searching for homologous sequence within sister chromatid and subsequent strand invasion to form displacement loop (D-loop). Domains found within each protein are shown. OB — oligosaccharide binding domain. Wh — winged-helix domain. NLS — nuclear localization signal. RING — really interesting new gene. BRCT — BRCA1 C terminus. Size of each polypeptide is indicated in aa.

Among the most well-studied process in nematode mitotic recombination is the formation of the RAD-51 presynaptic filament following DNA end resection. This step is aided by multiple factors either directly by helping RAD-51 to displace RPA bound to resected DNA or by stabilizing the nascent filaments. Once the presynaptic filament is established, RAD-51 engages with the template duplex to search for the homologous sequence. BRCA proteins (BRC-1 and BRC-2) and Rad51 paralogs facilitate the nucleation and assembly of RAD-51 nucleoprotein filaments on ssDNA (Figure 2). Mutations of their human counterparts, including BRCA1, BRCA2 and Rad51 paralogs confer breast and ovarian cancer predisposition in humans, as well as Fanconi anemia (FA) — a complex congenital disease associated with bone marrow failure and cancer predisposition [22, 23, 24].

### BRC-1/BRD-1 complex

*C. elegans* BRC-1 and BRD-1 proteins, like their human counterparts, interact directly with each other via their N-terminal RING finger domains [25]. As well as possessing C-terminal BRCT repeat domains, BRD-1 additionally contains three ankyrin repeats, which interact with the E2 ubiquitin-conjugating enzyme, LET-70 [26]. Biochemical and cellular studies have shown that BRC-1/BRD-1 complex possesses intrinsic E3 ubiquitin-ligase activity, which is activated in an ATL-1 and MRE-11-dependent manner in response to DNA damage [26]. While this activity catalyses the accumulation of poly-ubiquitylated species at sites of DNA damage, the proteins modified by ubiquitylation and the functional consequence remain unclear. In human cells, reports have suggested that human BRCA1-BARD1 directly binds RAD51 to stimulate homologous dsDNA capture *in vitro* [27]. More recent work also suggested that the BRCA1/BARD1-RAD51 interaction is important for stabilization of RAD51 at perturbed replication forks (RFs) to prevent fork degradation upon HU-treatment [28^•^]. In nematodes, a direct interaction between BRC-1/BRD-1 and RAD-51 has not been observed [25]. Yet, in meiosis, *brc-1* deletion delays RAD-51, but not RPA focus formation [29^•^,30^•^] indicating that it may perform a similar role in RAD-51 stabilization/loading as human BRCA1/BARD1. The nature of this role remains to be explored.

### BRC-2/(DSS-1?)

In human cells, PALB2 links BRCA1 to the recombination mediator protein, BRCA2 [31]. At present, a PALB2 ortholog has not been identified in *C. elegans*. Whether there is a bridging or direct interaction between nematode BRC-1 and BRC-2 remains unknown. In human cells, DSS1 interacts with BRCA2 and directly facilitates RPA displacement via a DNA mimicry-like mechanism [32]. Although co-expression of nematode DSS-1 with BRC-2 improves overall BRC-2 solubility [6], evidence for stable complex formation between DSS-1 and BRC-2 is lacking. Furthermore, DSS-1 addition does not have any effect on BRC-2’s strand annealing activity or its ability to stimulate RAD-51-mediated strand exchange in bulk assays [6] suggesting that DSS-1 does not perform a key role in HR in *C. elegans*. Despite this, nematode BRC-2 contains most of the critical domains found in the human homolog albeit at reduced size and number of domain repeats. These include a single BRC repeat, TR2 domain and DNA-binding domain composed of a single OB fold. Human BRCA2 is a much larger protein, which contains eight BRC repeats, a C-terminal DBD domain containing three OB folds, a C-terminal TR2 domain as well as multiple other regions that serve as protein-protein interaction hubs [33]. Importantly, a human ‘mini-BRCA2’ constructed as a fusion of one or two BRC-repeats, a C-terminal DBD and TR2 region is sufficient to largely complement loss of full-length BRCA2 [34] in mammalian cells as well as in strand exchange assays *in vitro* [32]. Thus, nematode BRC-2 represents a minimal functional RAD-51 loading factor, analogous to mammalian mini-BRCA2.

Initial studies revealed that BRC-2 interacts strongly with RAD-51 through its N-terminal BRC-TR2 domains and binds ssDNA, but not dsDNA [33]. This allows for specific RAD-51 targeting to RPA covered ssDNA regions post resection. Furthermore, BRC-2 was shown to inhibit RAD-51 ATPase activity [6]. Since ATP-bound RAD-51 is more stable on ssDNA than the ADP-bound form [35], this mechanistically explained how BRC-2 stabilizes nucleating RAD-51 clusters. The BRC-repeat and TR2 domain of BRC-2 display two modes of interaction with RAD-51. The BRC repeat binds RAD-51 monomers in solution and when injected into the worm germline is sufficient to strip RAD-51 from ssDNA, causing loss of RAD-51 foci. Conversely, the TR2 domain binds specifically to RAD51-ssDNA filaments and when injected into the worm germline, stabilizes and increases RAD-51 foci [36]. This led to the proposition that BRC and TR2 act as a RAD-51 loading unit, with the BRC repeat recruiting RAD-51 monomer and acting to transport a portion of the RAD-51 pool to damage sites and TR2 then acting as a stabilizing region to load a RAD-51 nucleus in a stable ATP-bound state on ssDNA. These nuclei should be then able to grow into nascent filaments. The activities of the BRC-2 domains and RAD-51 were subsequently confirmed for human BRCA2 peptides [35,37] and full-length human BRCA2 [38].

### RAD-51 and RPA

Once loaded on ssDNA, nematode RAD-51 (AAA+ ATPase protein) displaces trimeric ssDNA-bound RPA complex. While in other eukaryotes RPA consists of three subunits, the third small subunit of RPA has not yet been identified [20] in nematodes. Interestingly, the *C. elegans* genome encodes an RPA-2 paralog, RPA-4. Recent work has shown that, unlike RPA-1 and RPA-2 complex, RPA-4 does not play an essential role in DNA replication and meiotic HR, but localizes to sites of DNA damage and promotes germline apoptosis in *rpa-2* mutants [39]. RAD-51 forms helical nucleoprotein filaments similar to those of RecA or human RAD51 [13]. Interestingly, unlike yeast and humans where DMC1 — a meiosis-specific recombinase acts in meiotic prophase, no meiotic RAD-51 paralog is found in *C. elegans*. The difference in the sequence of the L1 DNA binding loop in DMC1 makes it highly tolerant to mismatches during strand exchange [15^•^], facilitating homeologous recombination. Intriguingly, nematode RAD-51 contains a similar motif in the L1 loop making it a mismatch tolerant recombinase like DMC1. A chimeric protein containing a ‘Rad51-like’-mismatch intolerant L1 loop displays low mismatch tolerance without compromised DNA strand exchange activity. However, this mutant completes meiosis without any apparent problems. These observations imply that perhaps the ability to tolerate mismatches is more strongly dictated by accessory factors rather than the intrinsic property of the core recombinase. Indeed, deletion of genes such as *brc-1*, *rtel-1*, *him-6 and mlh-1* were shown to increase the frequency of recombination between heterologous sequences (het-rec) in meiosis [40^•^], while deletion of *msh-2* and *msh-6* strongly supressed it. The mismatch intolerant RAD-51 variant only mildly supressed het-rec upon *brc-1* and *rtel-1* knockdown [15^•^]. It is possible that mismatch (in)tolerance plays a more important role during mitotic HR at perturbed RFs within regions containing DNA repeats.

### RAD-51 paralog-Shu complex (RFS-1/RIP-1/SWS-1)

A canonical RAD-51 paralog, RFS-1 is highly unstable protein on its own but forms a stable, soluble heterodimeric complex with the highly divergent paralog RIP-1 [13]. *In vitro*, the RFS-1/RIP-1 complex binds the 5′ end of RAD-51 filaments, stabilizes them and promotes DNA strand exchange [13,41]. At the interface of RAD-51 and RFS-1, a conformational change occurs, making RAD-51 units more ‘open’ and by proxy more proficient for DNA strand exchange. This is referred as RAD-51 ‘remodelling’ [13]. More recently, the use of single-molecule imaging tools facilitated the detailed biophysical analysis of the RAD-51 filament assembly process [14^••^]. RFS-1/RIP-1 was shown to dynamically engage with 5′ RAD-51 filament ends, stabilizing them by preventing catastrophic RAD-51 dissociation events. This allows RAD-51 filaments to grow faster in a 3′ to 5′ direction along the ssDNA axis. Timely dissociation of RFS-1/RIP-1 from filament ends is dependent on intrinsic ATPase activity of RFS-1. ATPase mutants of RFS-1/RIP-1 exhibited prolonged dwelling on RAD-51 5′ filament ends; this stabilizes RAD-51 on ssDNA, but hinders RAD-51 filament growth. The inclusion of purified BRC-2 in the same system increased RAD-51 nucleation rates. Overall, the presence of both RFS-1/RIP-1 and BRC-2 results in efficient RPA displacement due to combined stimulation of RAD-51 nucleation, stabilization and growth. *In vivo*, BRC-2 was also shown to acts as a RAD-51 nucleation factor upstream of RAD-51 paralogs, as previous proposed [42]. Whether BRC-2 binds and stabilizes the other (3′) filament end as was proposed for human BRCA2 [43] remains to be addressed. Previous reports also indicate that RFS-1 and the N-terminal domain of BRC-2 directly, but weakly interact [33]. The functional importance of this interaction remains unclear. The SWS-1 protein containing a conserved SWIM domain interacts with RIP-1 through its Walker B motif [44]. The precise role of SWS-1 is currently unknown. One possibility is that it may function in targeting RFS-1/RIP-1 to RFs or post-replicative gaps as proposed for yeast Shu complex and Rad55-Rad57 [45]. Finally, a postsynaptic function of RAD-51 paralogs stemming from synthetic lethality with HELQ-1 in meiosis [46] remains elusive. Human RAD51 paralogs were shown to directly interact with HELQ [47], but whether a similar interaction is conserved in *C. elegans* and its functional role is not known.

### Displacement loop formation and its processing

Following RAD-51 filament formation, RAD-51 facilitates the homology search and catalyses strand exchange resulting in the formation of a displacement loop (D-loop) intermediate. Homology search is likely assisted by RAD-54 [7]. Two critical helicases, RTEL-1 and HELQ-1, play a role downstream of RAD-51 filament formation in *C. elegans*. Both of these proteins operate in parallel pathways as evident by their synthetic lethal genetic interaction [46]. Specifically, the elevated meiotic crossover (CO) frequency in *rtel-1* mutants [48], D-loop disruption activity of purified RTEL-1 and its inability to disrupt Rad51 filaments [5], suggest a role in the synthesis-dependent strand annealing (SDSA) branch of HR, which yields non-crossover (NCO) products. Consistently, HR is altered in RTEL-1 deficient strains as assayed by an HR reporter assay [10]. The role of HELQ-1 is less well understood. HELQ disruption in mammalian cells reduces HR efficiency in DR-GFP reporter assay [47]. In addition, *helq-1*/*rfs-1* double mutant display strong synthetic lethality, due to impaired meiotic HR with persistent RAD-51 foci accumulating in late pachytene [46]. Biochemical data suggests that HELQ-1 can disrupt binding of RAD-51 on dsDNA, but not ssDNA [46]. Perhaps HELQ-1 could disrupt RAD-51 bound to dsDNA following strand invasion, which would allow for more efficient D-loop extension. However, the precise role of HELQ-1 in HR remains to be determined.

### Double Holliday junction formation and processing

The invaded strand within a D-loop configuration is extended by DNA synthesis. The extended strand could then be displaced to allow second-end capture to form a double-Holliday junction (dHJ), which must be resolved for proper chromosome segregation. This is achieved by multiple redundant pathways that process dHJs to complete repair. HIM-6 (BLM homolog) as part of the BTR complex, possesses HJ dissolution activity, which yields exclusively NCO products, which is evident by a synthetic lethal interaction between HIM-6 and RTEL-1 [5]. The second pathway to process dHJ is termed resolution, which can be catalysed by multiple nucleases (also known as resolvases). The group of resolvases consisting of GEN-1, HIM-18 (SLX-4, SLX4 homolog) and MUS-81 incise dHJ and produce both CO and NCO products. It is likely that HIM-18, MUS-81 & XPF-1 act in conjunction as part of the SMX complex, similar to human cells [49]. This is supported by a synthetic lethal interaction between *him-6* and *him-18* [4], *him-6* and *mus-81* [50] and *him-6* and *slx-1* [51]. GEN-1 and the SLX-4/SLX-1 complex also display similar DNA cleavage preferences as their human counterparts *in vitro*, although nematode GEN-1 also plays a part in DNA damage signalling *in vivo* [52]. Recently, a possible third pathway of late HR intermediate resolution mediated by the LEM-3 nuclease has been identified in *C. elegans* [53^••^]. It remains to be established whether LEM-3 or its human homolog ANKLE1 can resolve dHJ. Given that no combination of resolvase mutants blocks crossover formation entirely, there are likely additional processing activities that remain to be identified.

## HR plays a key role when DNA replication encounters DNA damage

In addition to conventional DSBs repair, HR can function in response to replication collision with DNA damage, such as lesions induced by hydroxyurea (HU), TOP1 poison — camptothecin (CPT), UV-C or DNA ICL-inducing agents — cisplatin (CDDP), nitrogen mustards or UVA-TMP. Interestingly, cellular responses to these agents also involves recruitment of HR factors but with several distinct features.

### Repairing replication-perturbing DNA damage

In Escherichia coli (*E. coli*), the majority of spontaneous recombination events occur at ssDNA gaps formed behind the replisome [54]. Similarly, BRCA2-deficient human cells accumulate ssDNA gaps behind the replisome when levels of endogenous DNA lesions are high [55]. Similarly, in *C. elegans*, unlike at conventional DSBs, *mre-11* and *atm-1* are dispensable for ATL-1 activation in response to replication-fork blockage by HU [21]. As RPA-coated ssDNA is critical for ATL-1 activation, RPA is likely already present at post-replicative gaps after fork perturbation (Figure 1b). RAD-51 is then recruited to perturbed forks where it presumably assembles onto ssDNA gaps as a filament. This is aided by mediator proteins similarly to conventional DSB repair. Interestingly, unlike BRC-2, the RFS-1/RIP-1/SWS-1 complex is dispensable for RAD-51 focus formation at IR-induced DSBs, but is required for RAD-51 focus formation after exposure to CPT, UV-C, CDDP and nitrogen mustards [42]. In accordance, RAD-51 paralog deficient worms are only slightly sensitive to IR but display strong sensitivity to CPT and CDDP. This indicates that RAD-51 paralogs are preferentially required to repair perturbed replication forks (RFs). The nematode strains lacking HELQ-1 [56] or RTEL-1 [5], which both function after RAD-51 filament formation, display higher sensitivity to RF-blocking and ICL-inducing agents, unlike BRC-1 [42]. Similarly, the SLX-1/SLX-4 complex appears to have a more important role during the response to replication-coupled DNA damage [51], while SLX-1/SLX-4/MUS-81 complex functions primarily in dHJ resolution during conventional DSB repair.

### DNA ICLs as a specific lesion requiring HR-mediated repair

ICLs stand out as a separate category of replication-perturbing DNA damage due to a preferential requirement for FA genes (Figure 1c). Among these, *C. elegans* FCD-2 is a critical factor acting downstream of ATL-1 and upstream of RAD-51 [57, 58, 59]. FCD-2 is mono-ubiquitylated in response to DNA damage and *fcd-2* deletion sensitizes worms exclusively to ICL inducing toxins. As described above, RFS-1/RIP-1/SWS-1 complex plays a preferential role in RAD-51 loading at ICLs rather than conventional DSBs [42]. Loss of HELQ-1 or RTEL-1 also confers strong sensitivity towards ICL-inducing agents [5,56]. Interestingly, mammalian HELQ was shown to be important for HR during ICL repair, in a pathway parallel to FANCD2 [47]. A similar observation was made in worms showing additive sickness between FCD-2 and HELQ-1 [56]. Since HELQ-1 is expected to act downstream of FCD-2, perhaps the FA pathway has multiple branches or genes involved might have roles other than in ICL repair. DOG-1 (FANCJ homolog) — a well characterized helicase, which participates in ICL repair downstream of RAD-51, also maintains polyCG tract stability during unperturbed DNA replication [42,58].

## Concluding remarks

The nematode model, owing to its inherent simplicity and ease of genetic and biochemical manipulation, has provided many unique discoveries concerning the regulation and execution of mitotic HR. History has proven many of these to be conserved in higher organisms, including mammalian systems. Insights gained from reconstitution of the nematode HR system *in vitro*, whole-organism monitoring of genome instability and multi-generational tracking of mutagenesis have the potential to spear-head our understanding of DNA double strand break repair at levels currently inaccessible to mouse models, mammalian cell biology and mammalian protein biochemistry. Future technical advances in high-throughput sequencing and ‘omic’ methods, optics and super resolution microscopy as well as biophysical methods and structural biology will undoubtedly fuel the potential of this simple, yet useful system to study recombination and DNA repair.

## Conflict of interest statement

Simon J. Boulton is also scientific co-founder and VP Science Strategy at Artios Pharma Ltd., Babraham Research Campus, Cambridge, UK.

## References and recommended reading

Papers of particular interest, published within the period of review, have been highlighted as:

• of special interest
•• of outstanding interest

## CRediT authorship contribution statement

**Ondrej Belan:** Writing - original draft, Visualization. **Roopesh Anand:** Writing - review & editing. **Simon J Boulton:** Writing - review & editing.
